# Postoperative infections after non-elective cesarean section – a retrospective cohort study of prevalence and risk factors at a single center in Denmark administering prophylactic antibiotics after cord clamping

**DOI:** 10.1186/s12884-022-05300-y

**Published:** 2022-12-17

**Authors:** Katja Kuhr, Paul Bryde Axelsson, Betina Ristorp Andersen, Ida Lise Arevad Ammitzbøll, Tine Dalsgaard Clausen, Ellen Christine Leth Løkkegaard

**Affiliations:** Department of Obstetrics and Gynaecology, Nordsjaellands Hospital Hillerød, Dyrehavevej 29, 3400 Hillerød, Denmark

**Keywords:** Puerperal infection/prevention and control, Surgical wound infection/prevention and control, Cesarean section, Antibiotic prophylaxis/standards, Guidelines as topic

## Abstract

**Background:**

Mothers giving birth by non-elective cesarean section have considerably higher risk of developing postoperative infection, than mothers giving birth by elective cesarean section. Meta-analyses have shown that the risk of infection is reduced when administering antibiotics at least 30 min prior to skin incision rather than after cord clamping. If given prior to incision, antibiotics are present in the neonatal bloodstream for up to 24 h after delivery, with early exposure to antibiotics potentially disturbing development of the gut microbiome. We aimed to retrospectively assess the prevalence of postoperative infection after non-elective cesarean section at a single labor ward administering antibiotics after cord clamping, additionally investigating risk factors for developing postoperative infections.

**Methods:**

In this retrospective cohort study, we included a total of 2,725 women giving birth by non-elective cesarean section in 2010–2017 with a review of records for prenatal risk factors, labor management, and perinatal outcomes. The primary outcomes were a main composite infection of development of either endometritis, surgical-site infection, or sepsis in conjunction with a relevant antibiotic prescription. Secondary outcomes included infection of unknown focus, mastitis, urinary tract infection, and pneumonia.

**Results:**

A total of 88 patients developed a main composite infection (3.2%). These infections subdivide into endometritis (*n* = 37/2725, 1.4%), surgical-site infection (*n* = 35/2725, 1.3%) and sepsis (*n* = 15/2725, 0.6%). We found a high body mass index (aOR = 3.38, 95%CI 1.93–5.92) and intrapartum fever (aOR = 2.26, 95%CI 1.22–4.59) to be independent risk-factors for developing postoperative infection after non-elective cesarean section. Furthermore, we found delivery by a more expedient emergency grade 2 cesarean section (aOR = 0.61 95%CI 0.37–0.998) compared to grade 3 to be a protective factor for developing postoperative infection after non-elective cesarean section.

**Conclusion:**

In a labor ward administering antibiotics after cord clamping at non-elective cesarean births, we find a low prevalence of main composite infections when compared to estimates from meta-analyses on the topic. We conclude that administration of prophylactic antibiotics after cord clamping appears to result in acceptable rates of postoperative infection and avoids transplacental-transmission of antibiotics to the infant.

**Supplementary Information:**

The online version contains supplementary material available at 10.1186/s12884-022-05300-y.

## Background

Mothers delivering by cesarean section (CS) have a five to twenty-fold higher risk of infections than mothers giving birth vaginally and surgical-site infections are more prevalent in CS, than other surgical procedures [1–4]. Administration of prophylactic antibiotics has the potential to significantly reduce the risk of postoperative infections [5]. When prophylactic antibiotics are administered at least 30 min prior to skin incision, the rates of endometritis and surgical-site infection can be reduced by 38–43% [5]. The prevalence of combined postoperative infections when antibiotics are given respectively prior to or after cord clamping ranges between 3.9 and 5.4% and 6.9–7.6%, respectively [1, 5]. However, none of the meta-analyses on this topic, distinguish the risk between elective and non-elective CS [1, 5]. Clinical studies have found a 1.5–2.4 times higher risk of surgical-site infection after non-elective CS compared to elective CS, presumably as a consequence of ongoing labor being common with contamination of bacteria from the birth canal and limited time for proper preparation of the patient or the surgeon [3, 6, 7]. Recent studies have implied that rates of serious maternal infections requiring contacts to a hospital are low when giving prophylactic antibiotics after cord clamping [8]. Even then, infections are manageable with oral antibiotic treatment in most cases.

The World Health Organization recommends administration of prophylactic antibiotics for any type of surgery within 60 min prior to skin incision, and because the national guideline for CS recommends prophylactic antibiotics prior to skin incision, most delivery wards in Denmark follow this practice [9, 10]. The timing according to cord clamping is important due to potential transmission of antibiotics to the infant. The antibiotic used prophylactically in Denmark, the second-generation cephalosporin cefuroxime, has been found to be present in the neonatal bloodstream for up to 24 h after birth [11]. Studies have implicated that there is a link between early exposure to antibiotics and a disturbance of the infant gut microbiome [12, 13] and possibly increased risk of asthma, eczema, and allergies [14–16].

Non-elective CS are distinctly different from elective CS with an inherent time constraint in the event of an obstetric emergency. As research regarding prophylactic antibiotics prior to or after umbilical cord clamping has mostly focused on elective CS, there is a knowledge gap on the absolute risk of infection for non-elective CS. Identifying certain women at greatest risk of infection, might also help women and clinicians to select the most pertinent treatment.

We aimed to retrospectively assess the prevalence of postoperative infection after non-elective CS at a single facility not administering antibiotics before cord clamping. Secondary objectives were to identify risk factors for main composite infections in non-elective cesarean section.

## Methods

### Study population

**Fig. 1:**
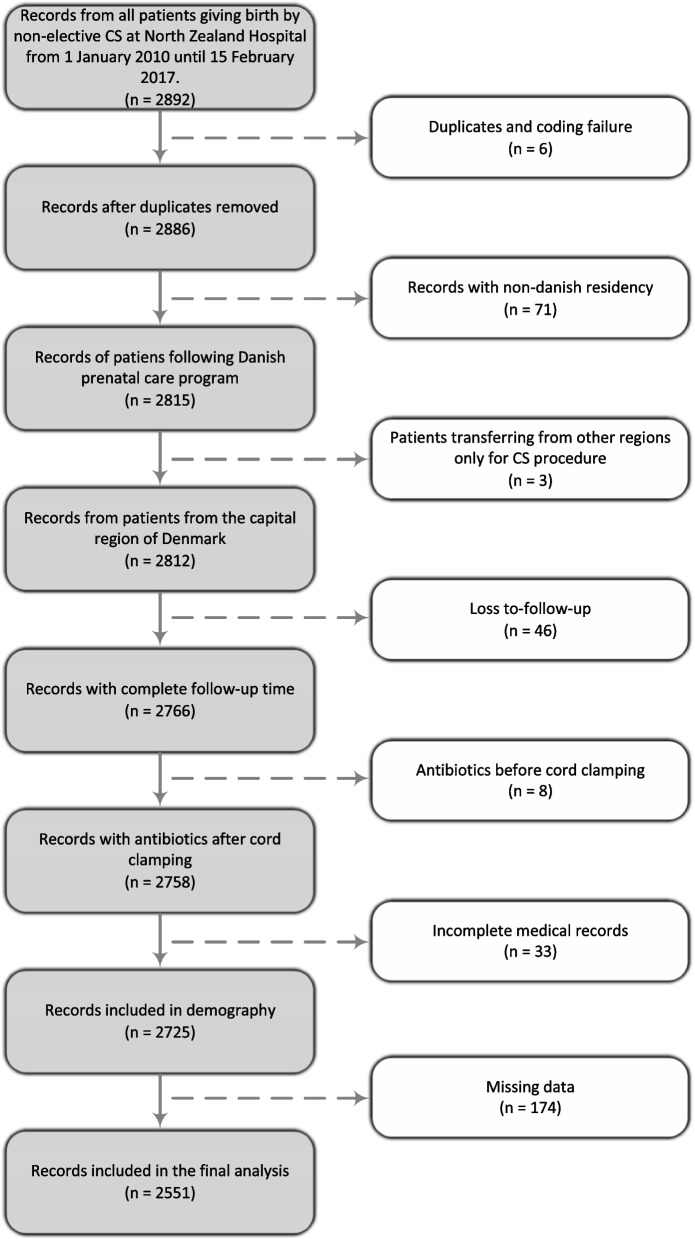
Exclusion and inclusion flowchart. Flowchart showing exclusion criteria and number of women excluded from the final analysis. Missing data includes data not available of the following categories: BMI, CS classification or previous CS, which can also be seen in Table 1

In this retrospective cohort study, electronic health records for all 2,892 women giving birth by non-elective CS at Nordsjællands Hospital, Hillerød, in the period from January 1st 2010 until February 15th 2017 were manually reviewed. Women were selected based on the Nordic Medico-Statistical Committee (NOMESCO) classification of surgical procedures for non-elective CS: KMCA10A, KMCA10D, and KMCA10E. Women who either were coded by mistake as having had non-elective CS or their health records being in duplicate were excluded. To decrease probability of unrecognized loss to follow-up, women of non-Danish residency and those transferring from other regions within Denmark to have their surgery at our labor ward were excluded. Women lost to follow-up were also excluded, such as those moving outside of the area our hospital covers, as well as women for whom the health records regarding discharge were not available. Women who received prophylactic antibiotics prior to cord clamping were excluded. After exclusion, 2,725 women were included for demographic outcomes. Of these, women with incomplete information in their health records regarding risk factors were further excluded for the risk factor analysis, leaving 2,551 women, see Fig. 1; Table 1.

**Table 1:** Background characteristics. Background characteristics of the 2725 women included in the prevalence estimate. ,CS: Cesarean section. N/A: Not Applicable. *Antibiotic treatment immediately prior to CS due to either intrapartum fever, prolonged rupture of membranes (> 18/24 hours), preterm prelabor rupture of membranes, preterm labor, group B streptococcus (GBS) urinary tract infection, earlier GBS urinary tract infection, urinary tract infection by another bacterial agent, any prelabor diagnosed infection, or previous delivery of an infant with GBS. ** Smoking in 3. Trimester

	Total		Intrapartum antibiotics^∗^		No intrapartum antibiotics	
	N (%)		N	%	N	%
Total	2725		619	22.7%	2106	77.3%
Smoking**						
Yes	260 (9.5%)		47	7.6%	213	10.1%
No	2332 (85.6%)		531	85.8%	1802	85.6%
Quit smoking	132 (4.8%)		41	6.6%	91	4.3%
Body Mass Index (kg/m^2^)						
< 18.5	91 (3.4%)		12	2.0%	79	3.8%
18.5-24.99	1517 (56.3%)		341	55.6%	1176	56.5%
25-29.99	642 (23.8%)		136	22.2%	506	24.3%
≥ 30	443 (16.5%)		124	20.2%	319	15.3%
N/A	32 (1.2%)		6	1.0%	26	1.2%
Maternal age (years)						
< 30	1016 (37.3%)		262	42.3%	754	35.8%
30-39.99	1532 (56.2%)		323	52.2%	1209	57.4%
≥ 40	177 (6.5%)		34	5.5%	143	6.8%
CS grade (time to delivery)						
1. Grade (< 15 min)	217 (8.0%)		30	4.8%	187	8.9%
2. Grade (< 30 min)	1067 (39.2%)		287	46.4%	780	37.0%
3. Grade (< 60 min)	1299 (47.7%)		274	44.3%	1025	48.7%
N/A	142 (5.2%)		28	4.5%	114	5.4%
Previous CS						
Yes	663 (24.3%)		89	14.4%	575	27.3%
No	2049 (75.2%)		527	85.1%	1522	72.3%
N/A	12 (0.4%)		3	0.5%	9	0.4%
Gestational Diabetes Mellitus						
Yes	132 (4.8%)		28	4.5%	104	4.9%
No	2593 (95.2%)		591	95.5%	2002	95.1%
Rupture of membranes						
Yes	2184 (80.1%)		584	94.3%	1600	76.0%
No	541 (19.9%)		35	5.7%	506	24.0%
Intrapartum fever						
Yes	382 (14.0%)		312	50.4%	71	3.3%
No	2343 (86.0%)		307	49.6%	2036	96.7%
Group B Streptococcus urinary tract infection						
Yes	78 (2.9%)		48	7.8%	30	1.4%
No	2647 (97.1%)		571	92.2%	2076	98.6%

Women were divided into two groups, depending on whether or not they had received intrapartum antibiotics for an intrapartum infection. Characteristics of the included women are presented in Table 1. For risk factor analysis both women exposed to intrapartum antibiotics and not, were pooled.

The surgical preparation, antibiotic and postoperative care regimens at Nordsjællands Hospital Hillerød can be read in the online supplementary.

### Outcomes

All primary and secondary outcome data were based on a manual review of the medical records, with the variables listed in Tables 1, 2 and 3 and a full list in the online supplementary.

**Table 2:** Postpartum infection after non-elective CS. Main composite infections and secondary infections given as number and prevalence. 95% CI = 95% pointwise confidence intervals

	Number	Prevalence	95% CI
Total (*n* = 2725)			
Main composite infection:	**87**	**3.2%**	**(2.6–4.0)**
Endometritis	37	1.4%	(0.9–1.9)
Surgical-site infection	35	1.3%	(0.9–1.8)
Sepsis	15	0.6%	(0.3–0.9)
Secondary infections:	**217**	**7.9%**	**(7.0–9.0)**
Unknown focus	35	1.3%	(0.9–1.8)
Mastitis	104	3.8%	(3.2–4.6)
Urinary tract infection	64	2.3%	(1.8 − 3.0)
Pneumonia	7	0.3%	(0.1–0.5)
Other infections	7	0.3%	(0.1–0.5)
Total infections	304	11.2%	(10.0–12.4)

**Table 3:** Risk of postpartum infection after non-elective CS. Logistic regression analyses for main composite infections given as odds ratios (OR) and pointwise 95% confidence intervals (CI) of 2551 complete records for main composite infections, comprising; endometritis, surgical-site infection and sepsis (the OR for those sub-outcomes can be seen in the online supplementary). * Smoking in 3. Trimester. GDM: Gestational Diabetes Mellitus, ROM: Rupture of Membranes, GBS UTI: Group B Streptococcus urinary tract infection, IP AB: Intrapartum Antibiotics

	Main composite infections *n* = 87			
	Crude		Adjusted	
	OR	95% CI	OR	95% CI
Smoking*	1.05	0.50–2.20	0.94	0.44–2.00
Body mass index (kg/m^2^)				
< 18.5	2.30	0.93–5.68	2.27	0.91–5.66
18.5-24.99	Ref			
25-29.99	**1.78**	**1.00–3.18**	1.78	0.99–3.19
≥ 30	**3.35**	**1.93–5.81**	**3.38**	**1.93–5.92**
Maternal age (years)				
< 30	Ref			
30-39.99	0.67	0.43–1.06	0.76	0.47–1.21
≥ 40	0.43	0.13–1.39	0.47	0.14–1.58
CS grade (time to delivery)				
< 15 min	0.61	0.24–1.55	0.67	0.26–1.72
< 30 min	0.65	0.40–1.05	**0.61**	**0.37–0.998**
< 60 min	Ref			
Previous CS	0.69	0.39–1.22	0.72	0.40–1.29
GDM	1.07	0.38–2.97	0.91	0.32–2.59
ROM	1.21	0.67–2.16	1.20	0.65–2.22
IP fever	1.64	0.95–2.84	**2.26**	**1.11–4.59**
GBS UTI	1.75	0.62–4.92	2.20	0.75–6.47
IP AB	1.13	0.67–1.89	0.62	0.31–1.22

The primary outcome was defined as a main composite infection, appearing within 30 days postpartum including; endometritis including cervicitis, surgical-site infection, or sepsis. A valid outcome necessitated both a diagnosis made by a physician and a relevant antibiotic prescription. Surgical-site infections included both superficial and deep infections, but were further subdivided into those requiring surgical revision or not. During the study period the diagnosis for sepsis was based on the Systemic Inflammatory Response Syndrome (SIRS) criteria [17]. An outcome of sepsis required at least two SIRS criteria to be fulfilled and there to be a clinical suspicion of an infection.

Secondary outcomes included; urinary tract infection, mastitis with or without abscess formation, pneumonia, and infection with unknown focus. Secondary infections were defined as a diagnosis by a physician and/or a related positive culture and prescription of a relevant antibiotic. Women presenting with either fever or increase in C-reactive protein but without an infectious focus or a specific infectious diagnosis, were classified as “infection of unknown focus”.

### Data analysis

For prevalence confidence intervals, we used the Wilson Score interval [18], and Pearson’s Chi-Squared for evaluating distribution of risk factors between women either receiving antibiotics prior to CS or not.

Certain factors have previously been associated with increased risk of postoperative infection after CS, such as obesity [7, 19–22], diabetes, and rupture of membranes [20, 22]. We have also included additional factors after thorough theoretical reflections regarding their possible predictive nature. In total, associations between 10 presumed risk factors and all main composite infections and secondary infections, were estimated using univariate logistic regression analysis providing crude odds ratio with 95% confidence intervals. In multivariate logistic regression analyses adjustment was made for the same 10 risk factors: Pre-pregnancy body mass index in kg/m^2^ according to the World Health Organization classification [23] (Underweight: <18.5, Normal weight: 18.5-24.99, Pre-obesity: 25-29.99, Obesity, all classes combined: ≥30), smoking during pregnancy (Yes/No/Quit smoking), maternal age at birth in years (< 30, 30-39.99, ≥40), group B Streptococcus urinary tract infection recorded during pregnancy (Yes/No), gestational diabetes (Yes/No), previous CS (Yes/No), intrapartum fever defined as temperature > 38 °C (Yes/No), rupture of membranes before CS (Yes/No), emergency grade related to CS (1. Grade: <15 min, 2. Grade: <30 min, 3. Grade < 60 min) in accordance with a national classification [24], intrapartum antibiotics (Yes/No). For further specifications on intrapartum antibiotics, see the online supplementary. Only women with complete data for all predictive variables were used in the regression analysis. Statistical calculation was performed in IBM SPSS Statistics Version 25.0, Armonk, New York.

## Results

The background characteristics are presented in Table 1. Of the 2,725 women that met the inclusion criteria, 642 (23.8%) were pre-obese, 443 (16.5%) were obese, 382 (14%) presented with intrapartum fever, 619 (22.7%) received antibiotics during labor for a number of reasons described in materials and methods, and almost 50% of the women had a CS urgency grade, where anticipated time from decision to delivery was below 30 min.

Of the total included women, 87 (3.2%, 95% CI 2.6-4.0) experienced one of the main composite infections within 30 days after delivery. The main composite infections subdivide into: Endometritis (*n* = 37/2,725, 1.4%), surgical-site infection (*n* = 35/2,725, 1.3%) and sepsis (*n* = 15/2,725, 0.6%), see Table 2. Of the 35 patients with surgical-site infection, 15 (*n* = 15/2,724, 0.6%) underwent surgical site revision. Additionally, one patient had a mild case of surgical-site infection, which was managed without antibiotic treatment and does therefore not count towards the outcome definition. Mastitis with or without abscess formation was the most common postpartum infection (3.8%), followed by an infection of unknown focus, urinary tract infection, pneumonia, and other infections, see Table 2.

The following were identified as significant risk factors for developing a main composite infection: Pre-obesity (OR = 1.78, 95%CI 1.0-3.18), obesity (OR = 3.35, 95%CI 1.93–5.81), and intrapartum fever (adjusted OR = 2.26, 95%CI 1.11–4.59) (Table 3). Endometritis was not predicted by any of the investigated risk factors, but obesity (OR = 6.51, 95%CI 2.88–14.72) and intrapartum fever (OR = 2.27, 95%CI 1.05–4.92) were significant predictors for surgical-site infection, and being underweight (OR = 10.31, 95%CI 2.06–51.55) and pre-obese (OR = 4.55, 95%CI 1.13–18.25) were predictors for developing sepsis (online supplementary Table S 1). Furthermore, we found that having a more expedient delivery by emergency grade 2 cesarean section (aOR = 0.61 95%CI 0.37–0.998) compared to grade 3, decreased the risk of a postoperative infection after non-elective cesarean section (Table 3).

The characteristics of patients with sepsis based on hospital charts are presented in the online supplementary Table S 2, along with the assumed focus of infection, which was established in 11 of the total 15 sepsis cases. In the online supplementary Table S 2, we also report which SIRS criteria were fulfilled. In three of the sepsis cases, the infection was present before the CS was performed and in two of these, the infection was the indication for the non-elective CS. Only 7 (47%) of the sepsis patients had a positive blood culture, 6 (40%) had a negative blood culture and 2 (13%) did not have a blood culture performed. Furthermore, four patients were admitted to the intensive care unit, but only one was related to surgical-site infection and the others were presumably unrelated to the CS.

Risk factors for developing a secondary outcome infection can be seen in the online supplementary Table S 3.

## Discussion

In this retrospective study, we found a prevalence of main composite infections after non-elective CS of 3.2%, where most cases were endometritis and surgical-site infection. We also found that a high BMI and intrapartum fever were strong risk factors for postpartum infections, especially surgical-site infection.

Meta-analyses have reported that 6.9–7.6% of women develop a postoperative infection after CS, when prophylactic antibiotics are given after clamping the cord, but these meta-analyses do not distinguish between elective and non-elective CS [1, 5]. When investigating the prevalence of postoperative infection after CS, the prevalence was 1.5–2.4 times higher for non-elective than for elective CS [3, 6, 7]. Thus the infection prevalence after non-elective CS should presumably be higher than that reported for unspecified CS [1, 5]. A recent study by Winther et al. with review of medical records of women giving birth by elective CS only, performed at our facility during a similar time period as the current study, found a combined infection prevalence of 2.1%, when administering antibiotics after cord clamping [8]. This could indicate that the sterility procedures, population, and the conditions at our facility may differ from those in previous studies. There have not been many randomized studies investigating this subject in northern European countries, but a large well conducted trial out of Austria found no benefit of prophylactic antibiotics before cord clamping [25]. Our results may therefore only be generalizable to countries with similar healthcare infrastructure as is in place in Denmark.

A Danish population study by Leth et al. including 32,468 women giving birth in another region of Denmark, found a prevalence rate of 5.6% for surgical-site infection for 3,984 women giving birth by non-elective CS, where antibiotics were administered after cord clamping [3]. However, we find a prevalence rate for surgical-site infection of 1.3%, which is considerably lower than the 5.6% reported by Leth et al. [3]. This could be due to discrepant outcome definitions as they included women with either a positive culture, or hospitalization/reoperation for surgical-site infection, or treatment with dicloxacillin after discharge and we included only women treated at the hospital. Supported by the findings from Ahnfeldt et al. as they in a prospective birth cohort of 1,871 women from another region find only half of the women with endometritis and none of the women with other types of infection were transferred to the hospital for treatment [26]. Therefore, we must consider that the total prevalence rate of endometritis and especially surgical-site infection may be underrepresented in our study. Likewise, Leth et al. report that postpartum infections are probably underreported, as 36–94% of infections are detected after discharge from the hospital [3]. However, infections treated in general practice are usually easily manageable and without severe health risks for the mother. In previous randomized trials regarding antibiotic timing, no efforts have been made to describe the severity of the cases.

In a previous study, we found that when pregnant women were asked if 2.4% rather than 4% absolute risk of postpartum surgical site infections and endometritis would make a difference to their choice of prophylactic antibiotic either before or after cord clamping respectively, 10 out of 14 women would opt for waiting until after cord clamping [27]. Therefore, it stands to reason that a prevalence rate of 3.2% for endometritis, surgical site infections, and sepsis would be found acceptable by most women. Even though our prevalence rate might be underestimated, the difference between women receiving antibiotics before vs. after cord clamping might not be as pronounced, due to the time constraints of an emergency CS, as we will discuss later.

We found a relatively high prevalence of sepsis, but only one fourth of sepsis patients were admitted to the intensive care unit, and of those only one woman had an infection focus from either endometritis or surgical-site infection. Severe sepsis can therefore be argued to have only been present in four patients, corresponding to a prevalence of 0.15%. The patients diagnosed with sepsis were on average admitted to the hospital for 8.4 days (see the online supplementary Table S 2 ), and the patients admitted to the intensive care unit, were on average admitted to the hospital for seven days in total, and not in intensive care the entire duration of the hospital stay. A recently published study, conducted at our facility for postpartum infections in elective CS only, when administering antibiotics after cord clamping, found a prevalence of sepsis of 0.06% and no admissions to the intensive care unit [8]. It is possible that our high prevalence of sepsis can be explained by a severe infection being the indication for performing the CS in the first place. Furthermore, non-elective CS in some cases are indeed urgent, in many cases with limited time for proper surgical preparation, leaving the patients at higher risk of surgical-site infection, possibly progressing into sepsis.

We found that overweight women had about twice the risk of main composite infection compared to normal weight women, especially due to increased risk of surgical-site infection and sepsis. These results are similar to findings from other studies [19–21]. Therefore, administration of prophylactic antibiotics prior to skin incision might be particularly indicated for overweight and obese women. Intrapartum fever was a risk factor for surgical-site infection in the current study, but as these patients usually receive intrapartum antibiotics, i.e. prior to skin incision, their a priori risk is difficult to determine. Surprisingly we did not find reported smoking to be a risk factor for any of the main composite infections, contrary to what has been described in earlier studies [28]. However, risk of pneumonia was significantly and greatly increased for smokers. Intrapartum fever and intrapartum antibiotics were risk factors for an infection of unknown focus, which may very well be associated with any of the main composite infections and indicate an underestimation of main composite infections in our study. None of the risk factors were associated with development of urinary tract infection, mastitis, or pneumonia, but in any case these infections are not influenced by the timing of prophylactic antibiotics [1, 5]. Intrapartum antibiotics were administered to 23% of the women in the cohort, and these women might be at an increased risk of an infection *a priori*, as evident by their different demographics. In effect, they were treated with antibiotics prior to cord clamping, and this might have lowered the rate of main composite infections in our study.

The classification of the non-elective CS into groups regarding time from decision to delivery showed that the risk of main composite infection was greatest for those delivering more than 30 min after the decision was made. This might indicate that delay in delivery for non-emergency situations, may increase the risk of infection, perhaps due to an ascending infection from the birth canal.

As women giving birth by non-elective CS are at a greater risk for developing postoperative infections, administration of prophylactic antibiotics prior to cord clamping might seem especially advantageous [7, 22]. However, we find that for almost 50% of the women undergoing a non-elective CS in our study, the anticipated time from decision to delivery was less than 30 min. Consequently, it would not be possible to administer prophylactic antibiotics at least 30 min prior to skin incision as the current evidence recommends. Therefore, the reduction in postpartum infection rate may be substantially lesser than found in randomized studies of elective CS. Further research regarding the effects of antibiotic prophylaxis prior to cord clamping in non-elective CS is needed. Administration of antibiotics takes time from the health care personnel that could instead be used to prepare the patient for other aspects of emergency surgery, where every minute can count for the infant’s survival and morbidity.

## Conclusion

For women giving birth by non-elective CS at a single delivery ward where prophylactic antibiotics are administered after cord clamping, we found a prevalence rate of 3.2% for hospital treated main composite infections, which is less than previously reported. Very few infections were serious. Women with a high BMI and intrapartum fever are at increased risk for developing main composite infection when administering antibiotics after cord clamping in non-elective CS. We conclude that administration of prophylactic antibiotics after cord clamping appears to result in acceptable rates of postoperative infection and avoids transplacental-transmission of antibiotics to the infant. The results of our study may only be generalizable to countries with similar healthcare infrastructure. An individual assessment of risk factors should be made in unison with the patient, taking time constrictions into account.

## Supplementary Information

**Additional file 1.**

## Data Availability

The datasets generated and analyzed during the current study are not publicly available as person-identifiable data may not be shared with the public under Danish law but specific anonymized data are available from the corresponding author on reasonable request.

## Abbreviations

CS: Cesarean section
OR: Odds ratio
aOR: Adjusted Odds ratio
SIRS: Systemic Inflammatory Response Syndrome
