# Supplementary material for: Postoperative infections after non-elective cesarean section – a retrospective cohort study of prevalence and risk factors at a single center in Denmark administering prophylactic antibiotics after cord clamping
Source: BMC Pregnancy Childbirth. 2022 Dec 17;22:945. doi: 10.1186/s12884-022-05300-y (PMC9758935; doi:10.1186/s12884-022-05300-y)
Supplement: Supplementary file 1 — Additional file 1. [file 12884_2022_5300_MOESM1_ESM.docx]

**Online Supplementary**

**Study population:**

*Study population:* Patient records were extracted from electronic health records, from women giving birth by non-elective CS at Nordsjællands Hospital, Hillerød, in the period from January 1^st^ 2010 until February 15^th^ 2017. All records (n=2,892) were reviewed and prenatal risk factors, labor management, and perinatal outcomes were recorded. Duplets (n=4) and misclassification of non-elective CS (n=2) were excluded from the dataset. Furthermore, non-Danish residents (due to lack of unique identification number) (n=71), records with missing entries (n=33), women with administration of antibiotics before cord clamping (n=8), and women transferred from hospitals outside the Capital Region Denmark, where electronic health records were not available (n=3) were excluded. A total of 167 records were excluded, see figure 1. Furthermore, 174 records were excluded for risk factor analysis, due to missing information on BMI, CS grade and/or previous CS.

**Definitions**

The variable *intrapartum antibiotics* (Table 3, Table S1, Table S3) comprised the following cases; prolonged rupture of membranes (>18/24 hours, guideline change in 2013), preterm prelabor rupture of membranes, fever during labor (≥38°C), group B streptococcus urinary tract infection, urinary tract infections caused by other bacterial agents, pneumonia, middle ear infection, gastroenteritis, sinusitis, throat infection, multi-resistant Streptococcus Aureus or a combination of the mentioned.

**Review of records and sampled variables**

For the review of records we collected data on: residency, maternal age at delivery, pre-pregnancy weight and height, gestational age, parity, previous CS, smoking status at birth, pre-pregnancy chronic illness (somatic and psychiatric), development of a pregnancy related illness (preeclampsia, gestational hypertension, gestational diabetes mellitus, group B streptococcus urinary tract infection or polymorphic eruption of pregnancy), use of medication during pregnancy and type of medication, whether a labor was induced or spontaneous, any type of infection during labor, intrapartum fever, intrapartum usage of antibiotics, indication and type of antibiotics, rupture of membranes at any time before CS, indication for performing CS, CS grade in accordance to urgency (22), whether antibiotics were given prior to or after clamping the cord, type of anesthesia, development of postpartum infection and type of infection. We also captured data on how many days after CS infection develops, postpartum body temperature if infection was present, whether postpartum infection was treated with antibiotics, whether patient is hospitalized due to infection and for how long, if patient is admitted to the intensive care unit, and infection of the child and admission on children’s ward

**Cases not included in the outcome:**

Besides the patients with a primary outcome described in the main article, two additional patients were diagnosed with endometritis, one diagnosed with sepsis, and one diagnosed with surgical-site infection, but these patients had an infection later than 30 days after delivery. Furthermore, a total of 22 cases of mastitis, one case of unknown focus, and one case of urinary tract infection, were diagnosed later than 30 days after delivery.

**Abdominal preparation regimen, antibiotic regimen and surgical dressing routines at the Nordsjællands Hospital, Hillerød:**

Before the cesarean section, a Foley catheter is inserted in the urinary bladder, and the skin cleaned with a povidone-iodine solution. Vaginal preparation is not performed. At the discretion of the surgeon, Pfannenstiel or Joel-Cohen incision, with a high preference for the latter, is performed on all patients. Thereafter, the incision is followed by the standard procedure: transverse lower uterine segment incision and delivery of the fetus and placenta. 1,5 grams of Cefuroxime (2nd generation cephalosporin) are given intravenously immediately after cord clamping, usually increased to 3 grams in case of BMI greater than 35 kg/m2. Suturing of the uterine incision is performed with or without exteriorization of the uterus. The abdominal wall is usually closed in two layers if subcutis is over 2 cm. Skin incisions are closed. The surgical dressing is recommended to be left untouched and unchanged for the first 3-4 days after the cesarean and preferably until the mother has left the hospital. The parents can call the maternity unit for up to 7 days postpartum should any complications or questions arise, after which contact to the primary care physician is advised and they can then refer patients back to the maternity ward.

|  |  | SIRS | | | | | Other characteristics | | | | | |
| --- | --- | --- | --- | --- | --- | --- | --- | --- | --- | --- | --- | --- |
| Cases | **Focus** | **Temp > 38°C** | **HR > 90** | **RF > 20** | **Leukocytes > 12** | **Sum of SIRS** | **Days after CS** | **ITA admission** | **Hospitalization (days)** | **Blood culture positive** | **Infection present before CS** | **General anesthesia** |
| 1 | Surgical-site infection | Yes | Yes | - | Yes | 3 | 15 | No | 5 | No | No | Yes |
| 2 | Surgical-site infection | Yes | Yes | No | - | 2 | 10 | No | 10 | No | No | No/Yes (reop) |
| 3 | Surgical-site infection | Yes | Yes | No | Yes | 3 | 1 | Yes | 1 | No | No | No/Yes (reop) |
| 4 | Possibly surgical-site infection | Yes | Yes | No | Yes | 3 | 10 | No | 7 | Yes | No | No/Yes (Reop) |
| 5 | Endometritis | Yes | Yes | - | - | 2 | 4 | No | 3 | Yes | No | No |
| 6 | Cervix infection | Yes | - | - | Yes | 2 | 1 | No | 18 | Yes | No | No/Yes (reop) |
| 7 | Urinary tract infection/pneumonia | Yes | Yes | - | Yes | 3 | 1 | Yes | 9 | Yes | No | No/Yes (reop) |
| 8 | Perforated appendicitis | Yes | Yes | Yes | Yes | 4 | 0 | Yes | 7 | No | Yes | Yes |
| 9 | Possibly urinary tract infection | Yes | Yes | - | - | 2 | 8 | No | 3 | Yes | Yes | No |
| 10 | Gastroenteritis | No | Yes | - | Yes | 2 | 0 | No | 16 | - | Yes | Yes |
| 11 | Tonsillitis | Yes | Yes | Yes | No | 3 | 21 | Yes | 11 | - | No | No |
|  |  |  |  |  |  |  |  |  |  |  |  |  |
| 12 | Unknown focus | Yes | Yes | Yes | No | 3 | 2 | No | 6 | No | No (MRSA) | Yes |
| 13 | Unknown focus | Yes | Yes | - | Yes | 3 | 2 | No | 17 | No | No | No |
| 14 | Unknown focus | Yes | Yes | - | Yes | 3 | 2 | No | 3 | Yes | No | No/Yes (reop) |
| 15 | Unknown focus | Yes | Yes | - | Yes | 3 | 1 | No | 10 | Yes | No | No |
|  |  |  |  |  |  |  |  |  |  |  |  |  |
| Total n (%) |  | 14 (87.5%) | 14 (87.5%) | 3 (18.8%) | 10  (62.5%) | N/A | Mean 5.2 | 4  (25%) | Mean 8.4 | 7  (43.8%) | 3  (18.8%) | 10  (62.5%) |

***Table S2: Characteristics of sepsis patients.*** *Women developing postpartum sepsis according to the SIRS criteria. Listed is the presumed focus of the sepsis, number of fulfilled SIRS criteria, and other characteristics relevant to the course of the infection. - : Unknown data, SSI: Surgical-Site Infection, HR: Heart Rate, RF: Respiratory Frequency, IRS: Systemic Inflammatory Response Syndrome, ITA: Intesive Care Unit, BMI: Body Mass Index, Reop: Reoperation due to bleeding or surgical-site infection, N/A: Not Applicable.*

|  | Unknown focus n=35 | | | | Urinary tract infection n=64 | | | | Mastitis n=104 | | | | Pneumonia n=7 | | | |
| --- | --- | --- | --- | --- | --- | --- | --- | --- | --- | --- | --- | --- | --- | --- | --- | --- |
|  | Crude | | Adjusted | | Crude | | Adjusted | | Crude | | Adjusted | | Crude | | Adjusted | |
|  | OR | 95% CI | OR | 95% CI | OR | 95% CI | OR | 95% CI | OR | 95% CI | OR | 95% CI | OR | 95% CI | OR | 95% CI |
| Smoking* | 1.30 | 0.45 - 3.74 | 1.38 | 0.47 - 4.04 | 0.99 | 0.42 - 2.32 | 0.87 | 0.36 - 2.08 | 0.59 | 0.21 - 1.64 | 0.56 | 0.20 - 1.55 | **12.74** | **2.83 - 57.24** | **13.15** | **2.59 - 66.84** |
| BMI (kg/m^2^) | | | | | | | | | | | | | | | | |
| < 18.5 | 0.63 | 0.08 - 4.78 | 0.73 | 0.10 - 5.66 | 0.95 | 0.29 - 3.13 | 0.85 | 0.25 - 2.83 | 0.51 | 0.12 - 2.14 | 0.49 | 0.12 - 2.05 | N/A |  | N/A |  |
| 18.5-24.99 | Ref |  |  |  | Ref |  |  |  | Ref |  |  |  | Ref |  |  |  |
| 25-29.99 | 1.42 | 0.64 - 3.14 | 1.46 | 0.65 - 3.28 | 0.99 | 0.52 - 1.86 | 0.96 | 0.50 - 1.83 | 0.81 | 0.43 - 1.50 | 0.83 | 0.45 - 1.55 | 1.13 | 0.21 - 6.18 | 1.15 | 0.19 - 7.06 |
| ≥ 30 | 1.25 | 0.49 - 3.22 | 1.18 | 0.44 - 3.12 | 1.47 | 0.78 - 2.78 | 1.49 | 0.77 - 2.89 | 1.02 | 0.53 - 1.98 | 1.11 | 0.57 - 2.16 | 0.83 | 0.09 - 7.46 | 0.89 | 0.09 - 8.56 |
| Maternal age (years) | | | | | | | | | | | | | | | | |
| < 30 | Ref |  |  |  | Ref |  |  |  | Ref |  |  |  | Ref |  |  |  |
| 30-39.99 | 0.62 | 0.30 - 1.26 | 0.74 | 0.36 - 1.54 | 0.76 | 0.45 - 1.29 | 0.79 | 0.46 - 1.37 | 1.01 | 0.60 - 1.68 | 1.02 | 0.61 - 1.72 | 0.33 | 0.06 - 1.81 | 0.35 | 0.06 - 2.08 |
| ≥ 40 | 0.69 | 0.16 - 3.01 | 0.91 | 0.20 - 4.09 | 1.50 | 0.64 - 3.52 | 1.51 | 0.63 - 3.64 | 0.88 | 0.30 - 2.56 | 0.92 | 0.31 - 2.71 | 1.38 | 0.15 - 12.42 | 1.61 | 0.15 - 16.99 |
| CS classification (time) | | | | | | | | | | | | | | | | |
| < 15 min | 1.66 | 0.46 - 6.01 | 1.84 | 0.50 - 6.74 | 0.73 | 0.26 - 2.08 | 0.72 | 0.25 - 2.07 | 0.73 | 0.26 - 2.08 | 0.70 | 0.24 - 2.00 | **18.43** | **1.91 - 178.00** | **25.03** | **2.36 - 265.00** |
| < 30 min | **2.15** | **1.02 - 4.54** | 1.80 | 0.84 - 3.86 | 0.97 | 0.58 - 1.64 | 1.08 | 0.63 - 1.86 | 1.13 | 0.68 - 1.86 | 1.17 | 0.70 - 1.95 | 3.71 | 0.39 - 35.72 | 4.62 | 0.46 - 46.65 |
| < 60 min | Ref |  |  |  | Ref |  |  |  | Ref |  |  |  | Ref |  |  |  |
| Previous CS | **0.19** | **0.05 - 0.81** | 0.25 | 0.06 - 1.08 | 0.64 | 0.33 - 1.23 | 0.57 | 0.29 - 1.12 | 0.66 | 0.35 - 1.23 | 0.63 | 0.33 - 1.20 | 1.22 | 0.24 - 6.28 | 2.14 | 0.35 - 13.31 |
| GDM | 2.05 | 0.62 - 6.82 | 1.99 | 0.57 - 6.91 | 0.66 | 0.16 - 2.73 | 0.52 | 0.12 - 2.23 | 0.30 | 0.04 - 2.19 | 0.29 | 0.04 - 2.13 | N/A |  | N/A |  |
| ROM | 3.99 | 0.95 - 16.72 | 2.37 | 0.55 - 10.25 | **0.50** | **0.29 - 0.85** | **0.51** | **0.29 - 0.90** | 0.96 | 0.63 - 1.75 | 0.91 | 0.49 - 1.70 | 0.34 | 0.08 - 1.51 | 0.37 | 0.07 - 2.08 |
| IP fever | **3.05** | **1.46 - 6.33** | 1.49 | 0.57 - 3.84 | 0.40 | 0.14 - 1.10 | 0.43 | 0.14 - 1.32 | 0.81 | 0.38 - 1.70 | 0.74 | 0.30 - 1.82 | 1.00 | 0.12 - 8.29 | 1.16 | 0.08 - 17.70 |
| GBS UTI | 1.02 | 0.14 - 7.55 | 0.75 | 0.10 - 5.77 | N/A |  | N/A |  | 0.49 | 0.07 - 3.55 | 0.50 | 0.07 - 3.74 | N/A |  | N/A |  |
| IP AB | **3.23** | **1.62 - 6.43** | 2.10 | 0.86 - 5.16 | 0.96 | 0.53 - 1.75 | 1.02 | 0.49 - 2.12 | 0.89 | 0.49 - 1.61 | 0.95 | 0.46 - 1.99 | 1.35 | 0.26 - 6.97 | 2.70 | 0.32 - 22.67 |

**Table S3: Risk of postpartum infection after non-elective CS.** Logistic regression analyses for secondary infections given as odds ratios (OR) and pointwise 95% confidence intervals (CI) of 2551 complete records for secondary infections, comprising: unknown focus, urinary tract infection, mastitis and pneumonia. * Smoking in 3. Trimester, BMI: Body Mass Index, GDM: Gestational Diabetes Mellitus, ROM: Rupture of Membranes, GBS UTI: Group B Streptococcus urinary tract infection, IP AB: Intrapartum Antibioti
